# Susceptibility Profiles of *Helicoverpa armigera* (Hübner) (Lepidoptera: Noctuidae) to Deltamethrin Reveal a Contrast between the Northern and the Southern Benin

**DOI:** 10.3390/ijerph16111882

**Published:** 2019-05-28

**Authors:** Eric Tossou, Ghislain Tepa-Yotto, Ouorou K. Douro Kpindou, Ruth Sandeu, Benjamin Datinon, Francis Zeukeng, Romaric Akoton, Généviève M. Tchigossou, Innocent Djègbè, John Vontas, Thibaud Martin, Charles Wondji, Manuele Tamò, Aimé H. Bokonon-Ganta, Rousseau Djouaka

**Affiliations:** 1 International Institute of Tropical Agriculture (IITA), 08 P.O. Tri-Postal, Box 0932 Cotonou, Benin; g.tepa-yotto@cgiar.org (G.T.-Y.); d.kpindou@cgiar.org (O.K.D.K.); b.datinon@cgiar.org (B.D.); romaricakoton88@gmail.com (R.A.); tchigossougenevieve@yahoo.fr (G.M.T.); m.tamo@cgiar.org (M.T.); 2 Direction of Plant Production, University of Abomey-Calavi, P.O. Box 526 Abomey-Calavi, Benin; aimehbg@gmail.com; 3 School of Crop and Seed Production, National University of Agriculture (UNA-Benin), P.O. Box 43 Kétou, Benin; 4 Faculty of Science, Department of Biochemistry, University of Yaoundé I, P.O. Box 812 Yaoundé, Cameroon; sandeu2009@yahoo.com (R.S.); zeusfranck07@yahoo.com (F.Z.); 5 Life And Earth Sciences, National University of Science, Technology, Engineering and Mathematics, P.O. Box 2282 Abomey, Benin; djegbe1@yahoo.fr; 6 Institute of Molecular Biology and Biotechnology, Foundation for Research and Technology, N Plastira 100, 70013 Crete, Greece; vontas@imbb.forth.gr; 7 Pesticide Science, Agricultural University of Athens, Ieraodos 75, 11855 Athens, Greece; 8 Cirad UR Hortsys, Université Montpellier, Campus international de Baillarguet, 34 398 Montpellier, France; thibaud.martin@cirad.fr; 9 Biosciences Unit, University Houphouet Boigny, Cocody 01 BP 6483Abidjan, Côte d’Ivoire; 10 Vector group, Liverpool School of Tropical Medicine, Liverpool L3 5QA, UK; charles.wondji@lstmed.ac.uk

**Keywords:** *H. armigera*, pyrethroid resistance, Benin

## Abstract

*Helicoverpa armigera* is an indigenous species in Africa and has been reported in the destruction of several crops in Benin. Management of *H. armigera* pest is mainly focused on the use of synthetic pyrethroids, which may contribute to resistance selection. This study aimed to screen the susceptibility pattern of field populations of *H. armigera* to deltamethrin in Benin. Relevant information on the type of pesticides used by farmers were gathered through surveys. Collected samples of *Helicoverpa* (F_0_) were reared to F_1_. F_0_ were subjected to morphological speciation followed by a confirmation using restriction fragment length polymorphism coupled with a polymerase chain reaction (RFLP-PCR). F_1_ (larvae) were used for insecticide susceptibility with deltamethrin alone and in the presence of the P450 inhibitor Piperonyl Butoxide (PBO). Deltamethrin and lambda-cyhalothrin were the most used pyrethroids in tomato and cotton farms respectively. All field-sampled *Helicoverpa* were found to be *H. armigera*. Susceptibility assays of *H. armigera* to deltamethrin revealed a high resistance pattern in cowpea (resistance factor (RF) = 2340), cotton (RF varying from 12 to 516) and tomato (RF=85) farms which is a concern for the control of this major polyphagous agricultural pest. There was a significant increase of mortality when deltamethrin insecticide was combined with piperonyl butoxide (PBO), suggesting the possible involvement of detoxification enzymes such as oxidase. This study highlights the presence of P450 induced metabolic resistance in *H. armigera* populations from diverse cropping systems in Benin. The recorded high levels of deltamethrin resistance in *H. armigera* is a concern for the control of this major agricultural pest in Benin as the country is currently embarking into economical expansion of cotton, vegetables and grain-legumes cropping systems.

## 1. Introduction

The cotton bollworm, *Helicoverpa armigera* (Hübner) (Lepidoptera: Noctuidae) is a key polyphagous pest infesting several crops such as cotton, tomato, maize, sorghum, chickpea, pigeon pea, pulses, vegetables and tomato crops worldwide [1]. This pest causes an estimated loss of over US$ 5 billion annually in Africa despite application of pesticides [2]. In contrast to many other arthropod pests, *H. armigera* has a wide geographical range of host plants and can adapt to new environments [3]. New publications on *H. armigera* in South America [4,5] created suspicions of the presence of other species of *Helicoverpa* in Africa given the proximity between the two continents. Species identification remains complex with possibilities of misidentification due to morphological similarities between *H. armigera* and *Heliothinae* species (such as *Helicoverpa zea* (Boddie)) [6]. The restriction fragment length polymorphism (RFLP)-PCR method is used to determine a genetic variation between the populations. PCR-RFLP amplifies specific partial regions of the mitochondrial DNA (mtDNA) genome followed by the digestion of PCR products with specific endonucleases to distinguish between combining patterns of partial regions of the mitochondrial gene [7]. This technique has been optimised for confirming *H. armigera* [7].

Economic damage caused by *H. armigera* is very significant worldwide [2]. Over the past decades, management of *H. armigera* has become increasingly difficult due to its high reproductive and damage potentials [8]. The strong tendency of *H. armigera* to move from one fruit to another without consuming it completely, explains why this pest causes extensive damages to crops even when the number of larvae is relatively low [9].

Synthetic insecticides have shown promising control of chewing and sucking insect pests in the early 1980s, these compounds are applicable to *Helicoverpa* pests. Cotton and tomato, the main *Helicoverpa* host plants, are generally protected from pest damage by application of insecticides such as carbamates, organophosphates and synthetic pyrethroids [10,11]. In comparison to carbamates and organophosphates, synthetic pyrethroids are effective at low dosages for controlling *H. armigera* at extremely low cost [10,12]. Excessive and continuous applications of pyrethroid insecticides for *H. armigera* control initiated since the mid-1980s in most countries have led to resistance selection pressure in several field populations of this insect [10,13,14]. With this development of insecticide resistance, the control of *H. armigera* has become critical in many regions worldwide [8,15]. Recent studies have reported the increased resistance of *H. armigera* to pyrethroids in Pakistan [16], South India [17], Spain [18] and West Africa [19]. Durigan et al. [20] pointed to metabolic resistance mechanisms and showed that the quantities of cytochrome P450 (CYP337B1, CYP337B2 and CYP337B3) are greater in resistant strains compare to susceptible strains after exposure to deltamethrin. Furthermore, Martin et al. [21] also demonstrated that P450 enzymes contribute to deltamethrin resistance in *H. armigera* populations from West Africa. These results were further confirmed by Brun-Barale et al. [19] who identified the modified P450 genes. Insecticide resistance development usually occurs with the appearance of genetic mutations and/ or increased enzymatic detoxification [20,22]. Recent studies indicate that metabolic resistance is primarily responsible for pyrethroid resistance in *H. armigera*. Elevated oxidative detoxification (P450-based resistance) was confirmed as the major resistance mechanism to pyrethroids in Australia and Asia [20,23,24].

Very little is known about the susceptibility profiles of *H. armigera* to the commonly used agricultural insecticide deltamethrin across agro-ecological areas in Benin. Although many studies have been conducted worldwide on *H. armigera* [1,12,17,19,24], very few of these research works have been carried out in the western part of Africa [11,19,21]. In Benin for example, no study had extensively and simultaneously mapped the insecticide resistance profiles of this pest on several cropping systems such as cotton, vegetable and grain legumes currently under intensification. In addition to this lack of information on resistance mapping in Benin, no research had attempted to provide information on potential mechanisms conferring observed phenotypic resistance profiles in several cropping systems. Taking into account the current nationwide economic options made by the Benin government to promote the development of cotton, vegetables and grain legumes farming, there is a great need to regularly document and update information on insecticide resistance profiles and related resistance mechanisms developed by *H. armigera* nationwide. Data from this research will support strategic policies on the selection of cost-effective insecticides for better control of this polyphagous pest. This research documents on the control of *H. armigera* pest in various host plants, provides information on the extent of *H. armigera* resistance to deltamethrin in the South-North transect of Benin, investigates possible role of detoxifiers (P450 enzymes) in recorded phenotypic resistances. Information generated will help to improve decision making on the type of insecticides to be used for *H. armigera* control in different host crops and in different localities in Benin.

## 2. Materials and Methods

### 2.1. Study Sites

**Figure 1:**
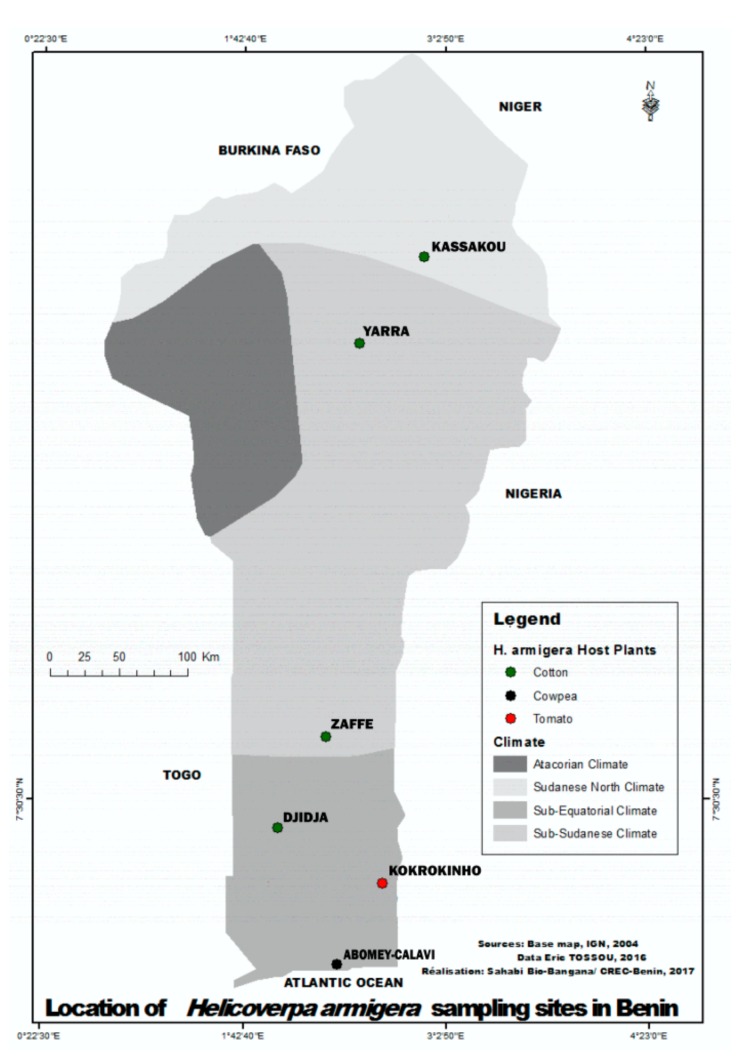
Map of Benin showing localities where *H. armigera* populations were found.

Benin is located between the Equator and the Tropic of Cancer at latitudes ranging from 6°30’ N to 12°30’ N and longitude ranging from 1° E to 3°40’ E. This country shares boundaries with Togo in the West, Burkina Faso and Niger in the North, and Nigeria in the East. Four main agro-ecological zones are found in the country. The North-Sudanese, the Atacorian, the Sub-Sudanese and the Sub-Equatorial climatic zones. A total of 6 agricultural settings were surveyed in these agro-ecosystems. Field populations of *Helicoverpa* spp. were sampled in the following 6 localities. Kassakou in the North-Sudanese area, characterized by one long dry season and a short rainy season, with relatively low humidity and rainfall (800 to 1000 mm per year), and high temperatures (up to 45°C during dry seasons). The localities of Yarra and Zaffé both in the Sub-Sudanese area with a long rainy season and a short dry season. Rainfalls ranging between 900 and 1200 mm, less hilly localities with wet savanna vegetation types. Finally, three localities (Djidja, Kokrokinho, Abomey-Calavi) in the Sub-Equatorial area that spans the southern part of the country and extends up to coastal areas of Benin. Two rainy and two dry seasons are recorded in these localities. The relative humidity is high, temperatures are relatively low, and the vegetation is a mosaic of coastal wetlands, forest, and wet savanna (Figure 1).

### 2.2. Insecticide Utilisation by Farmers in Surveyed Localities

The knowledge of farmers on the use of chemical pesticides for *H. armigera* control was assessed in surveyed tomato, cotton, and cowpea farms from November 2016 to November 2017. Relevant information on the use of chemical pesticides for *H. armigera* control were gathered through focus group discussions, direct field observations and in-depth interviews. Information collected included: the types of pesticides used by farmers, the concentrations/doses applied and the application frequencies. Based on the number of farmers in each farm, we determined the minimal acceptable size of farmers to be interviewed in each locality. A total of 180 volunteer farmers consented this survey in the six studied localities.

### 2.3. Insects Sampling, Molecular Speciation and Insecticide Susceptibility Testing

#### 2.3.1. Laboratory Strain

A susceptible strain of *H. armigera* (SoucheVrac Sensible, SVS) was obtained from the Entomology laboratory of the Agricultural Research Institute for Development in Cameroon (IRAD-Garoua, Cameroon). SVS was transferred to the International Institute of Tropical Agriculture (IITA-Benin) insectary at pupae stage, and was reared following laboratory conditions and steps described by Nibouche [25]. This strain was used as the standard susceptible colony during insecticide susceptibility assays.

#### 2.3.2. Field Sampling of Wild Populations of Helicoverpa

Field populations of *Helicoverpa*were collected during rainy seasons in 6 agricultural localities namely (Kassakou, Yarra, Zaffé, Djidja, Kokrokinho and Abomey-Calavi) (Table 1; Figure 1). Collected samples of *Helicoverpa* (F_0_) were reared to F_1_. The parents (F_0_) were preserved in suitable containers filled with ethanol (100%), and stored at −20°C. These samples (F_0_) were later subjected to molecular speciation using RFLP-PCR [7]. F_0_ progenies (third instar larvae) were used for insecticide susceptibility assays (topical assays) with deltamethrin alone and in the presence of the P450 inhibitor piperonyl butoxide (PBO). Insects collected in the field were introduced into cylindrical plastic cups (4cm diameter; 5cm height) with a cover punched with holes to allow ventilation for breathing of larvae. Each cup was filled with 4g of solid agar media for feeding larvae throughout their transportation from the field to the IITA-Benin insectary.

#### 2.3.3. Laboratory Rearing of H. Armigera

Both laboratory and field strains of *H. armigera* were reared at 25±1°C, 75% Relative Humidity (RH), and a photoperiod of 12:12 Light:Darkness (L:D) in the insectary as described by Nibouche [25]. Briefly, larvae were reared on an artificial diet composed of maize flour (120 g/L), brewer yeast (40 g/L), white cowpea flour (172 g/L), honey (20 g/L), sorbic acid (2 g/L), ascorbic acid (6 g/L), agar (25 g/L), 40% formaldehyde (2 mL/L), erythrocin (0.05g/L) and acyclovir (1.2g/L). The fourth instar larvae were obtained and individualized into cylindrical plastic cups containing artificial diet (4g) to avoid cannibalism. Pupae were collected, disinfected and, morphological identifications were carried out before emergence of adults. Emerging adult males and females from F_0_ larvae were pooled for matting. Eggs from this matting were incubated for hatching and, larvae (F_1_) from hatched eggs were placed on artificial diet and reared to L_3_-larva stage; the developmental stage used for insecticide susceptibility assays (Topical assays) with deltamethrin insecticide.

#### 2.3.4. Molecular Speciation of Sampled Helicoverpa spp.

PCR-RFLP was used in this speciation. Total genomic DNA was extracted from sampled F_0_ individuals (parents) after they had produced their progenies (F_1_). Samples (F_0_) were analysed for molecular speciation and for predicting the species profiles of offspring to be submitted to insecticide susceptibility tests. Total DNA was obtained after crushing the whole insect and following the extraction protocoldescribed by Livak [26]. Extracted DNA was quantified using a nanodrop 8000 (Thermo Scientific, Mississauga, ON, Canada) and a working DNA solution of 40–50 ng/µL was prepared for amplification. Amplifications were carried out in 50µL reaction containing cytochrome oxidase I (COI) and cytochrome b (Cytb) (Table 2) according to Behere et al. [7].

DNA amplification was confirmed by running 5µL of the post-PCR products on 1.5% agarose gel stained with 1% Midori green. Two restriction enzymes (BstZ17I and HphI) were used for discriminating *H. armigera* species. The pattern of product sizes on the agarose gel was used for identifying *H. armigera* species following Behere et al. protocol [7] (Table 3).

#### 2.3.5. Insecticide Susceptibility Assays (Topical Assays)

As documented in most published papers, this assay was conducted on larval stages as they are the most cropsdamaging stages [9]. The selection of larvae for insecticide susceptibility analysis was supported by several published papers and standards [17,27]. Technical grade of deltamethrin 99% (Sigma-Aldrich, Taufkirchen, Germany) was used in this assay. Susceptibility of third instar larvae of *H. armigera* (F_1_ offspring) to deltamethrin was tested using topical application technique [10,27]. Two-fold serial dilutions were prepared in pure acetone according to Kranthi [28] and the following concentrations were obtained for bioassays with the susceptible strain: (0.01454; 0.02908; 0.05816; 0.1163; 0.2326; 0.4653; 0.9306 µg/g). A log-probit dose (dose-mortality) curve was drawn from this strain and a diagnostic dose (DD) of 2181µg/g was obtained. Five concentrations thrice replicated of the technical grade (0.01DD, 0.1DD, DD, 10DD, and 100DD) and a control with no insecticide (0DD) were then prepared for bioassays with the field strains of *H. armigera* (144 to 349 larvae). Each exposed larva had a body weight between 30–40 mg. One microliter of deltamethrin was applied topically to the pro-thoraxic dorsum of the pest. Larvae were tested individually in plastic cups (4cm diameter; 5cm height) containing 4g of artificial diet and punched at the top. Pure acetone was used as control. The mortality was assessed every 24 h post exposure and was monitored for a total of 72 h for both test and control samples. Larvae were considered dead when unable to move if prodded with a blunt probe or brush [10,27].

### 2.4. Synergist Test with Piperonyl Butoxide (PBO)

For this assay, 135 to 233 larvae of *H. armigera* from each locality were pre-exposed to the P450 inhibitor piperonyl butoxide 90% (PBO) (20 µg/µL) for one hour and immediately after, these larvae were exposed to deltamethrin at five concentrations thrice replicated of the technical grade (0.01DD, 0.1DD, DD, 10DD, and 100DD) so as to cover the full dose range bioassay. Acetone solution was added to serve as the control solution. The diagnostic concentration was used for plotting the graph showing the effect of the PBO synergist on larvae. Larval mortality was monitored every 24 h after exposure until 72 h.

### 2.5. Data Analysis

Mortality curves for different tested concentrations of deltamethrin were computed using probit analysis (WinDL50software; CIRAD, Montpellier, France). Results were expressed as percentage mortalities. Abbott’s formula was used for correcting the recorded mortalities. Resistance factors (RFs) were determined as the ratio of the lethal dose for 50% (LD_50_) of field collected populations of insects and the susceptible strain. RFs were used to classify levels of insecticide resistance as: susceptible (RF = 0–1), low resistance (RF = 2–10), moderate resistance (RF = 11–30), high resistance (RF = 31–100), very high resistance (RF > 100) following descriptions made by Torres-Vila et al. [18]. The level of significance was set at *p* < 0.05.

## 3. Results

### 3.1. Insecticides Used for Cotton and Tomato Farming in Benin

**Figure 2:**
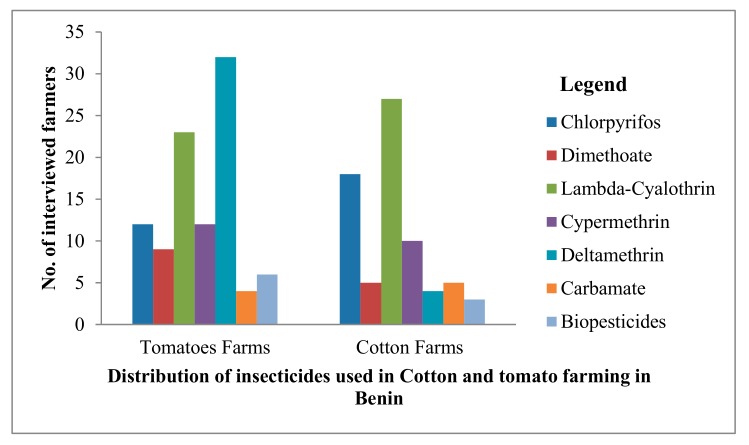
Insecticide use in cotton and tomato farming in Benin.

We conducted interviews and group discussions with a total of one hundred and eighty farmers (140 for interviews and 40 for group discussions) from tomato and cotton farms to identify insecticide families used for *H. armigera* control. Different classes of insecticides were identified namely: pyrethroids, organophosphates, carbamates and biopesticides (Bacillus thuringiensis, Neem) (Figure 2).

Synthetic pyrethroids (deltamethrin, lambda-cyhalothrin and cypermethrin) were identified as the main insecticides (60%) used against *H. armigera*. Deltamethrin and lambda-cyhalothrin were the most used pyrethroids in tomatoes farming (50.9%) and cotton farming (28.7%) respectively. Organophosphates (chlorpyrifos and dimethoate) were also mentioned by farmers and used either as single or in combinations with pyrethroids. Information provided by farmers revealed that tomato farms are sprayed once every 10 days, whereas cotton farms are treated two times per month. Despite the use of these insecticides, cases of plants and fruits attack by insects were still reported by cotton and tomato farmers, raising the failure of these synthetic insecticides for tomato and cotton plants protection.

### 3.2. Molecular Validation of Morphologically Identification of Helicoverpaarmigera.

**Figure 3:**
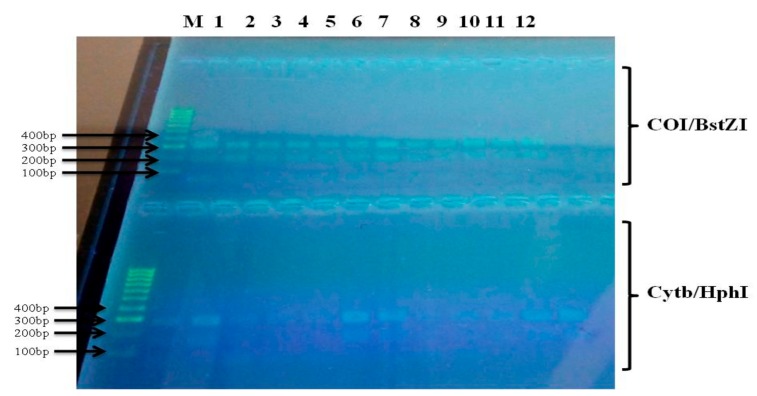
Agarose gel electrophoresis of PCR products digested with BstZ17I and HphI restriction enzymes, showing different restriction fragment length polymorphism (RFLP) patterns of analysed samples of *H. armigera*. M: molecular weight markers. Lanes 1-2, 3-4, 5-6, 7-8, 9-10 and 11-12:*H. armigera* specimens from Kassakou, Zaffé, Kokrokinho, Djidja, Abomey-calavi and Yarra respectively.

COI and Cytb genes fragments were successfully amplified in one hundred and twenty *Helicoverpa* samples. 20 samples of F_0_ adult were analysed from each surveyed agricultural setting. Following digestions of amplified PCR products with restriction endonucleases (BstZ17I and HphI). All analysed samples from the 6 surveyed localities were identified using molecular techniques as *H. armigera* (Table 3 and Figure 3).

### 3.3. Bioassays

#### 3.3.1. Susceptibility of *H. armigera* to Deltamethrin

Following susceptibility assays conducted with deltamethrin, the LD_50_ of the susceptible strain of *H. armigera* (SVS) was 0.064 µg/g (Table 4), while the diagnostic dose (DD: LD_99.9_) was 2.181 µg/g. When field samples (L_3_ from the F_1_) were exposed to same serial dilutions of deltamethrin, lower mortality rates which correspond to high levels of resistance were observed. Mortality rates of 9.7% and a LD_50_ = 149.780 µg/g were recorded with *H. armigera* from the Abomey-calavi farms; mortality rates of 14.36% and LD_50_ = 20.378 µg/g recorded with *H. armigera* from the Zaffé farms; mortality rates of 31.77% and LD_50_ = 36.16 µg/g for samples from Djidja; mortality rates of 34.35% and LD_50_ = 5.428 µg/g for samples from Kokrokinho; mortality rates of 43.3% and LD_50_ = 2.105 µg/g for samples from Yarra; mortality rates 52.9% and DL_50_ = 0.800 µg/g for *H. armigera* from the Kassakou farm (Table 4).

**Figure 4:**
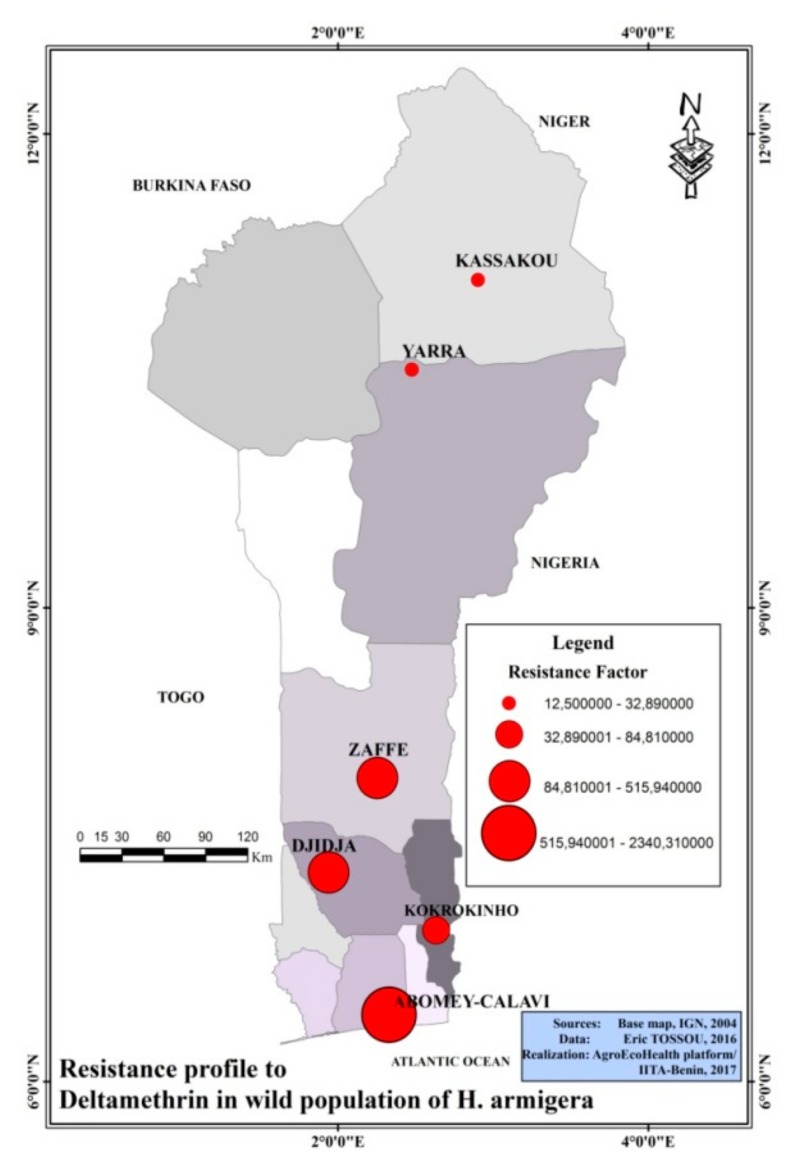
Map of Benin showing the resistance profile of *H. armigera*populations to deltamethrin in the North-South transect. The resistance profile is expressed in term of resistance factor (RF) calculated from the DL_50_.

Determined Resistance Factors (RF) from recorded mortalities were 12.5; 32.89; 318.41; and 565 folds higher for Kassakou, Yarra, Zaffé and Djidja cotton farms respectively, as compared to the susceptible laboratory strain SVS (Figure 4). For *H. armigera* collected in tomato farms, RF was 84.81 folds higher (samples from Kokrokinho) than the susceptible strain “SVS”. As for samples from cowpea farms of Abomey-Calavi, we recorded a RF of 2340.31 folds higher than the susceptible strain “SVS” (Figure 4). Overall, the results of this research revealed higher levels of deltamethrin resistance in cowpea farms. We also recorded an increased resistance pattern as we move down from the North to the southern part of Benin (Figure 4).

#### 3.3.2. Mortality Rates of *H. armigera* When Exposed to Combinations of PBO Synergist and Deltamethrin

**Figure 5:**
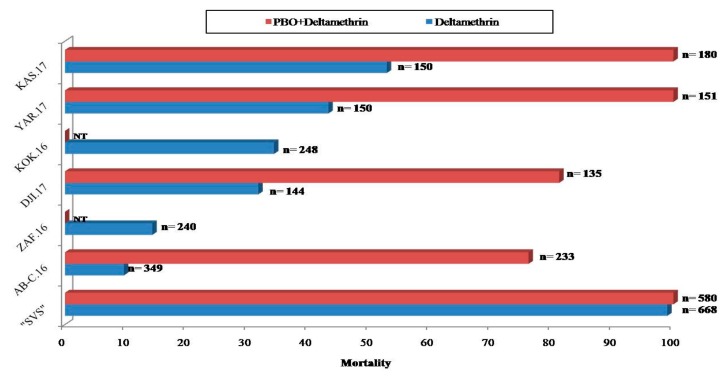
Recorded mortalities of *H. armigera* populations following exposures to diagnostic dose of deltamethrin in the presence or absence of piperonyl butoxide (PBO) synergist. *n* = number of tested larvae (third instar), NT = No tested.

When PBO was combined with deltamethrin insecticide, the recovery of susceptibility was observed for all *H. armigera* tested (Table S1). The susceptibility was total for *H. armigera* populations from cotton farms of Kassakou and Yarra with mortality rates reaching 100% (Figure 5). With populations from the cotton farm of Djidja, 81.2% mortality was recorded with the combination PBO and deltamethrin whereas, 76.2% mortality was observed with *H. armigera* populations from the surveyed cowpea farm of Abomey-Calavi (Figure 5). Resistance profiles recorded in cotton farms (North of Benin) were mostly metabolic whereas in cowpea and tomato farms (South of Benin) several mechanisms which include increase activities of oxidase detoxification were observed. No synergist test was performed on *H. armigera* from Zaffé and Kokrokinho farms due to the low number of larvae collected in these localities.

## 4. Discussion

### 4.1. The Use of Insecticide for H. armigera Control

This study revealed that up to 60% of tomato and cotton producers in Benin use pyrethroids to control *H. armigera* in their farms. Deltamethrin and lambda-cyhalothrin are the main insecticides used against *H. armigera* in tomato and cotton production respectively. In cotton farming systems, Lambda-cyhalothrin is most often combined with Chlorpyriphos- ethyl for *H. armigera* control. Data from this survey also revealed that despite the newly approved national Integrated Pest Management (IPM) strategy which replaces the use of deltamethrin by either lambda cyhalothrin (pyrethrinoid) or chlorpyriphos-ethyl (organophosphate) [29], many farmers continue to actively use deltamethrin for fighting *H. armigera* in cotton fields. As recorded in tomato farming, Decis 12,5 CE (deltamethrin) is also intensively used by cowpea farmers (Frequency: 6 days/1L/ha) [30]; this could explain high resistance profiles observed in *H. armigera* populations harvested in cowpea farms.

### 4.2. Molecular Identification of H. armigera

Molecular speciation using COI and Cytb targets coupled with digestion by BstZ17I and HphI respectively helped confirming the presence of *H. armigera*in the 6 surveyed sites in Benin. Previous studies based on morphological characters such as forewings of noctuid moths have shown several limitations for differentiating some members of *Helicoverpa* family. In this research, we have used for the first time in Benin a published DNA based protocol [7] for confirming the presence of *H. armigera* in different host plants in the North-South transect of the country. This molecular based identification of *H. armigera* in addition to traditional morphological methods described by Matthews [31] constitute effective combinations for improved speciation of *H. armigera* and better control of this polyphagous pest.

### 4.3. Susceptibility of Helicoverpa armigera to Deltamethrin in Benin

IPM strategies implemented in most cropping systems in Benin are mainly based on the use of synthetic chemicals such as pyrethroids, organochlorines and organophosphates [11]. Of these insecticide families, two pyrethroids (deltamethrin and lambda-cyhalothrin) are mostly used for protecting tomato and cotton crops against the polyphagous *H. armigera* as reported in Benin and other West African countries [32,33,34,35]. This regular use of pyrethroids by farmers against this pest of high economic interest has less been backed by a constant monitoring of their susceptibility patterns to insecticides. This research revealed that farmers are continuing to use deltamethrin for *H. armigera* control despite the fact that this insect has already developed high resistance levels to this insecticide. This study highlights the need to provide evidence-based information to farmers on the resistance profiles of the main agricultural pests to guide their insecticide usage. Data generated revealed a high resistance of *H. armigera* to deltamethrin in cowpea farms (RF = 2340) followed by tomato farms (RF = 84.81) and finally cotton farms (RF ranged 12.5 to 515.94). This high resistance to deltamethrin could be attributed to the overuse and miss-use of this insecticide during many decades in vegetable (tomato), grain legumes (cowpea) and cotton fields across the country [14,33,34]. In addition to the misuse and overuse of insecticides by farmers, the poor quality of pesticides used for pest control has also been documented as favouring the selection of resistance in wild population of pests [36,37]. Resistance levels of *H. armigera* populations from cotton fields was lower than previously reported in other localities of Benin [11,19] and, in some west African countries such as: Burkina Faso and Ivory Coast [29]. The relatively low resistance recorded in cotton farms could be related to recent modifications made nationwide on IPM strategies such as the alternative use of insecticide families for cotton pest control [29]. In Benin for example, the treatment of cotton plants starts with two spraying of organophosphate followed by a combination of pyrethroids and organophosphate during the last four spraying passages. Such alternative use of different families of insecticides make difficult the development of high resistance levels in surveyed cotton farms. In Contrast, alternative treatments are neither conducted in tomato farms nor in cowpea cropping systems hence, the relatively high resistance profiles recorded with *Helicoverpa* populations from these two commodities. In tomato and cowpea farms, deltamethrin remains the insecticide of choice for *H. armigera* control [30,35]. Resistance selection observed could be primarily due to the permanent use of this insecticide by farmers or, through use of poor quality deltamethrin for pests control as documented by some authors [33,34]. Very surprisingly, we recorded high resistance levels in the cotton farms of Djidja and Zaffé in the South Benin; contradicting data from the surveyed cotton farms of Kassakou and Yarra in the North Benin. As a matter of fact, both cotton farms of Djidja and Zaffé are surrounded by tomato, and cowpea farms which are regularly under pyrethroid treatments. It is possible that *Helicoverpa* populations of Djidja/Zaffé cotton farms in addition to receiving insecticides residues from cotton treatments also get exposed to pyrethroid insecticides leaching from surrounding tomato and cowpea farms during rainfalls. This combined selection pressure from cotton insecticides and leached insecticides from neighbouring tomato and cowpea farms could explain the higher resistance levels recorded in cotton farms of Djidja and Zaffé contrary to other cotton farms surveyed in the northern Benin (Figure 4). High resistance profiles were globally recorded in the Southern part of Benin compare to North. These high resistance profiles of insects in the Southern Benin had also been documented by Djouaka et al. [38] and other scientists working on insecticide resistance in malaria mosquitoes [36,37,39]. These high resistance levels recorded in southern Benin, compared to northern part of the country, could be associated to combinations of resistance selection factors such as the wide range of agrochemicals misused and overused by farmers, and other environmental pollutants (xenobiotics) which are still to be well identified. The overuse and misuse of pyrethroids lead to a loss of effectiveness of this insecticide as most pests will gradually develop resistance to the selection pressure; this will further increase the financial cost of pests treatment. This misuse of insecticides also leads to environmental pollution causing the imbalance of ecosystems [40]. The importance of conducting insecticide resistance studies on *H. armigera* resides on the current nationwide need of boosting cotton, vegetables and grain legumes production for increased communities income in Benin. Maps on insecticide resistance spread generated will support strategic policies on the selection of more effective insecticides for better control of this polyphagous pest on different cropping systems and in different localities in Benin.

### 4.4. Resistance Mechanisms Developed by H. Armigera Populations to Deltamethrin

When field populations of *H. armigera* were exposed to PBO synergists prior to deltamethrin bio-assays, we recorded a significant increase in observed mortalities; implying the consistent involvement of P450-mediated detoxification enzymes in observed resistance mechanisms. These results are similar to those reported by Durigan et al. [20] in Brazil; Achaleke et al. [41] and Brun-Barale et al. [19] in Central Africa and West Africa respectively. However, the fact that mortalities below 100% were recorded in Djidja and Abomey-Calavi even in the presence of synergist PBO may indicate the possible presence of other mechanisms of resistance in these *H. armigera* populations. Previous studies had revealed the involvement of esterase enzymes in highly resistant populations of *Helicoverpa* spp. from farms under IPM [42,43]. This research further highlights the implication of several families of metabolizers in recorded pyrethroid resistance in *H. armigera* populations in Benin. The effectiveness of synergists as recorded in this research suggests a possible combination of PBO and pyrethroids for improved performance of current treatments of cotton, vegetable and grain legumes farms in Benin. More investigations are needed for a better understanding of developed resistance mechanism pathways as these evidences are necessary for a better management of this pest and for reducing related economic losses.

## 5. Conclusions

This research focused on the use of synthetic chemicals in diverseagro-ecosystems forimproved pest control strategies. Results from this study revealed high levels of pyrethroid resistance in several populations of *H. armigera* in the North- South transect of Benin. An increasing resistance pattern was observed as we moved from the North to the southern Benin. A resistance contrast was also recorded in between populations of *H. armigera* from cotton, tomato and cowpea. The involvement of P450s metabolizers in the observed deltamethrin resistance was documented. Further investigation is being conducted into: assessing insecticide resistance profiles of *H. armigera* to other insecticides, screen for developed resistance mechanism pathways and study related fitness costs. This scientific evidence isimportant for the development of more tailored, cost effective and sustainable IPM strategies against this highly polyphagous pest.

## Figures and Tables

**Table 1:** Geolocalization of surveyed localities for *H. armigera* sampling.

Collection Site	Region (Agro Ecosystem)	Host Plant	Latitude Longitude	Sample Strains
Zaffé	Central (Sub-Sudanese)	Cotton	07°55.343’	ZAF.16
			002°15.256’	
Kokrokinho	South (Sub-equatorial)	Tomato	06°57.377’	KOK.16
			002°37.868’	
Abomey-Calavi	South (Sub-equatorial)	Cowpea	06°25.260’	AB-C.16
			002°19.684’	
Kassakou	North (North-Sudanese)	Cotton	11°4.520’	KAS.17
			002°54.127’	
Yarra	North (Sub-Sudanese)	Cotton	10°30.447’	YAR.17
			002°28.569’	
Djidja	Central (Sub-equatorial)	Cotton	07°19.495’	DJI.17
			001°56.393′	
Benoue and Mayo Rey	North	Cotton	-	‘SVS’
			-	

**Table 2:** Primers used for amplifying *H. armigera*sequences.

Primers		Primer Sequences
COI	COI-F02	5′CTC AAA TTA ATT ACT CCC CAT C′3′
	COI-R02	5′GGA GGT AAG TTT TGG TAT CAT T3′
Cytb	Cytb-F02	5′GAA TCC TTT AAT TTA AAA TAT AC3′
	Cytb-R02	5′AAA TAT GGG TTA GTT AAA GTT AA3′

**Table 3:** Expected sizes of digested PCR products for *H. armigera* identification.

Extraction	Amplifications	Digestion Enzymes	Digestion Products (pb)
*Helicoverpa* spp. DNA	Amplified product (COI)	BstZ17I	318 + 193
	Amplified product (CYTB)	HphI	280 + 154

**Table 4:** Recorded LD_50_ with laboratory and field samples of *H.armigera* when exposed to Deltamethrin.

Strain	Host Plant	Region (Agro Ecosystem)	*n*	LD_50_ (µg/g) (95% FL)	Slope ± SE
‘SVS’	Cotton	North	668	0.064 (0.035–0.064)	1.91 ± 2.29
KOK.16 (Kokrokinho)	Tomato	South (Sub-equatorial)	248	5.428 * (2.62–8.81)	1.02 ± 0.75
ZAF.16 (Zaffe)	Cotton	Central (Sub-Sudanese) South (Sub-equatorial)	240	20.378 * (12.88–34.07)	1.10 ± 1.44
KAS.17 (Kassakou)	Cotton	North (North-Sudanese)	150	0.800 * (0.132–4.85)	0.61 ± 0.06
YAR.17 (Yarra)	Cotton	North (Sub-Sudanese)	150	2.105 * (0.236–18.7)	0.56 ± 0.181
AB-C.16 (Abomey-Calavi)	Cowpea	South (Sub-equatorial)	349	149.780 * (31.12–720.84)	0.76 ± 1.65
DJI.17 (Djidja)	Cotton	Central (Sub- equatorial)	144	36.16 * (1.23–181.16)	0.32 ± 0.48

*N* = number of tested larvae (third instar), LD_50_ = dose that kills 50% of the tested sample, * = LD_50_ significantly different from that of the susceptible ‘SVS’ strain; 95% FL = Fidicial limits (95%).

## Supplementary Materials

The following are available online at https://www.mdpi.com/1660-4601/16/11/1882/s1, Table S1: LD_50_ for deltamethrin with PBO on susceptible and resistant strains of *H. armigera* in Benin.
