# Supplementary material for: Susceptibility Profiles of Helicoverpa armigera (Hübner) (Lepidoptera: Noctuidae) to Deltamethrin Reveal a Contrast between the Northern and the Southern Benin
Source: Int J Environ Res Public Health. 2019 May 28;16(11):1882. doi: 10.3390/ijerph16111882 (PMC6603559; doi:10.3390/ijerph16111882)
Supplement: Supplementary file 1 [file ijerph-16-01882-s001.pdf]

Supplementary table S1: LD_{50} for deltamethrin with PBO on susceptible and resistant strains of *H.armigera* in Benin

Strain	Host plant	Insecticides	n	LD_{50} (µg/g) (95%FL)	Slope±SE
"SVS"	Cotton	PBO+Delta	580	0.061 (0.036-0.07)	0.70±2.29
AB-C.16 (ABOMEY-CALAVI)	Cowpea	PBO+Delta	233	0.0003 (1.210-6-7.210-4)	0.44 ±1.28
ZAF.16 (ZAFFE)	Cotton	PBO+Delta	NT	–	–
DJI.17 (DJIDJA)	Cotton	PBO+Delta	135	0.136 (0.0488-0.3)	1.002±0.87
KOK.16 (KOKROKINHO)	Tomate	PBO+Delta	NT	–	–
YAR.17 (YARRA)	Cotton	PBO+Delta	151	0.0005 (2.210-6-6.810-4)	0.35±1.50
KAS.17 (KASSAKOU)	Cotton	PBO+Delta	180	0.032 (0.007-0.078)	0.81±1.20

n= number of tested larvae (third instar), LD_{50}= dose that kills 50% of the tested sample; 95%FL = Fidicial limits (95%); RF (Resistance factor) = LD_{50} of the tested strain/LD_{50} of the susceptible strain "SVS"; NT= No tested, Delta= Deltamethrin, PBO= Piperonyl butoxide.
